# Modelling the In Vitro Growth of Phytopathogenic Filamentous Fungi and Oomycetes: The Gompertz Parameters as Robust Indicators of Propolis Antifungal Action

**DOI:** 10.3390/jof9121161

**Published:** 2023-12-03

**Authors:** Catarina Passão, Cristina Almeida-Aguiar, Ana Cunha

**Affiliations:** 1 Department of Biology, University of Minho, Campus de Gualtar, 4710-057 Braga, Portugal; catarinaazvd@hotmail.com; 2 CBMA—Centre of Molecular and Environmental Biology, University of Minho, Campus of Gualtar, 4710-057 Braga, Portugal

**Keywords:** biological pesticides, filamentous phytopathogenic fungi, Gompertz model, phytopathogenic oomycetes, Portuguese propolis

## Abstract

Propolis is a resinous mixture produced by honeybees, mainly from plant exudates. With a rich chemical composition including many phenolic compounds, mostly responsible for its biological properties, namely antimicrobial ones, propolis may be a promising alternative to synthetic pesticides. The study of propolis from the south of Portugal and of its potential against phytopathogenic agents are still very recent and different methodological approaches hinder a comparison of efficacies. In this context, we aimed to test the value of a mathematical model for the multiparametric characterization of propolis’ antifungal action on solid medium assays. An ethanol extract (EE) of a propolis sample harvested in 2016 from Alves (A16) was characterized in terms of phenolic composition and antimicrobial potential against five phytopathogenic species. A16.EE (500–2000 µg/mL) inhibited the mycelial growth of all the species, with *Phytophthora cinnamomi* and *Biscogniauxia mediterranea* being the most susceptible and *Colletotrichum acutatum* being the least affected. The Gompertz mathematical model proved to be a suitable tool for quantitatively describing the growth profiles of fungi and oomycetes, and its parameters exhibit a high level of discrimination. Our results reveal that propolis extracts may have potential applications beyond traditional uses, particularly within the agri-food sector, allowing beekeepers to make their businesses more profitable and diversified.

## 1. Introduction

It is estimated that global food production would need to increase by 60% to feed the 9.8 billion people projected for 2050 [1,2]. Considering the limited availability of arable land, farmers worldwide will need to enhance production per hectare on existing agricultural lands [3]. On the other hand, an estimated 20–40% of crop yield is lost because of pests and diseases worldwide, with a huge impact on the sector and the global economy [4,5]. About 70–80% of crop losses derived from microbial diseases are caused by fungi [6,7,8,9], with more than 10,000 species of phytopathogenic fungi being known [10]. Worldwide, 4.6 million tons of synthetic pesticides are applied each year [11], and about 1000 active substances are used in its composition, 30% of which are highly poisonous to the environment [12,13]. Moreover, it has been estimated that less than 0.1% of the pesticides reach their target organisms, while the remainder contaminate the surrounding environment [14,15]. These harmful effects together with the growing interest in environmentally friendly approaches has increased the demand for biologically based viable alternatives [16]. Furthermore, in the context of the European Green Deal, the European Commission proposed binding targets to reduce the use of chemical pesticides and of the more hazardous pesticides by 50% by 2030 [17].

Propolis is a natural resinous product made by honeybees (particularly *Apis mellifera* L.) from plant exudates such as lipophilic materials on leaves and leaf buds, resins, mucilages and lattices [18,19], which are mixed with salivary enzymes (e.g., *β*-glucosidase) and bee wax [20]. More than 850 composts have already been identified in propolis from different regions, belonging to such diverse chemical classes as flavonoids, phenylpropanoids, terpenes, stilbenes, lignans and coumarins [21,22]. Their abundance depends upon the flora at the collection site and on the collecting season [18,19,20]. Propolis research over the last decades has proved that it has a broad range of biological properties such as antibacterial, antifungal, antiviral, antioxidant, (anti)genotoxic and antitumoral activities, closely related to its richness in phenolic compounds [20,23,24,25,26,27]. Therefore, propolis is a natural product with biopesticide potential [28,29]. Portuguese propolis is generally included in the poplar European temperate type [26], produced from poplar exudates, mainly *Populus nigra* L., rich in flavonoid aglycones (flavones and flavanones), phenolic acids and their esters [20,25,30,31,32,33,34]. However, Portugal exhibits a great botanical diversity and two different types of propolis were identified: a common temperate propolis type, observed in the north, central coast and Azores, with a high phenolic content, and a less common propolis, with lower phenolic content and an unusual composition in quercetin and kaempferol glycosides, characteristic of the central interior and south, with *Cistus ladanifer* L. being the main botanical source content [35,36,37]. Despite the growing demand for propolis-based products in several industries (like pharmaceuticals, cosmetics and food), the Portuguese supply is limited and unstandardized, and still not recognized as profitable or even useful by the majority of the beekeepers [38,39].

Anthracnose caused by *Colletotrichum* spp. is one of the most limiting diseases to the olive sector. *Colletotrichum acutatum* J.H. Simmonds is one of the main detrimental factors because it affects fruits at maturity, leading to significant poor fruit and oil quality and consequently significant yield losses [40]. *Colletotrichum fioriniae* (Marcelino & Gouli) R.G. Shivas and Y.P. Tan is also pathogenic, but less virulent [41,42]. The charcoal and bot canker diseases, caused by *Biscogniauxia mediterranea* (De Notaris) O. Kuntze and *Diplodia corticola* A.J.L. Phillips, A. Alves and J. Luque, respectively, cause high economic losses due to the development of symptoms in cork oak trunks that affect cork production and quality [43,44]. The oomycete *Phytophthora cinnamomi* Rands, responsible for ink disease, is a major soilborne pathogen of the European chestnut [45]. Considering the antifungal potential of propolis and the high incidence of the diseases described above in economically important crops of the Mediterranean basin, but of worldwide relevance, this study aims to evaluate the potential of a propolis sample from the south of Portugal against those five phytopathogenic species, envisaging an alternative to chemical synthetic fungicides.

Mathematical models used to predict microbial growth have been developed based on liquid cultures data, while models describing growth on solid surfaces are scarce [46]. Also, the quantitative characterization of mycelial growth is providential to better study the effect of antifungal agents in toxicological assays. We propose the use of a re-parameterisation of the Gompertz model [47] to describe the growth of filamentous fungi and oomycetes colonies on solid media (PDA), also evaluating the power of its parameters as indicators of toxicity, in this case assisting in the characterization of the antifungal and anti-oomycetal activity of a propolis extract against different phytopathogenic species. The Gompertz growth function describes a sigmoidal growth curve and is one of the most widely used nonlinear models in many aspects of biology, having been fitted to the growth of animals and plants as well as to tumour, bacterial and fungal growth, among others [47,48,49,50]. We aim to contribute with knowledge on the use of propolis in agronomic applications, an approach which has been rarely explored so far, while validating the use of the Gompertz model as an important tool in antifungal and anti-oomycetal assays, and ultimately for the valorisation of Portuguese propolis.

## 2. Material and Methods

### 2.1. Chemicals

All the chemicals were of analytical grade purity. Sodium carbonate (Na_2_CO_3_), aluminium chloride (AlCl_3_) and quercetin were obtained from ACROS Organics (Thermo Fisher Scientific, Bridgewater, NJ, USA). The Folin–Ciocalteu reagent, gallic acid and sodium molybdate (Na_2_MoO_4_) were purchased from Sigma-Aldrich (Merck KGaA, Darmstadt, Germany). The PDA medium (potato extract 4 g/L, dextrose 20 g/L and agar 15 g/L) was supplied from Thermo Scientific (Thermo Fisher Scientific Inc., Waltham, MA, USA). Ethanol was supplied by Carlo Erba Reagents (DASIT Group, Milan, Italy). Deionized water was treated in a Milli-Q water purification system (Millipore, Merck KGaA, Darmstadt, Germany).

### 2.2. Propolis Samples Origin and Extraction Procedure

The propolis sample was collected in June 2016 from a Portuguese apiary located in the Baixo Alentejo region, Alves, Beja district (N 37°35′31.2288″ N; O 7°35′54.1248″). It was very elastic and gluey, presenting a dark green colour. The sample designated by A16 was extracted with absolute ethanol (gummy, as previously reported [33]). Briefly, 15 g of raw propolis was added to 80 mL of ethanol (EtOH) and maintained under orbital agitation (110 rpm) for 24 h at room temperature in the dark. The resulting solution was filtered and the solid fraction was subjected to another cycle of extraction and filtration. The filtrates were then mixed and dried in a rotavapor (45 rpm, 40 °C), generating the ethanol extract A16.EE, which was stored at 4 °C until further use. The weight of the raw propolis sample (*W_S_*) and of the dried ethanol extract (*W_EE_*) were used to determine the yield of extraction (%) with Equation (1):

(1) $$\mathrm{yield}\;\left(\%\right)={{\;W}_{EE}/W}_{S}\;\times \;100$$

### 2.3. Chemical Characterization of A16.EE

#### 2.3.1. Total Phenolic Compounds Content

The total phenolic compounds content (TPC) was determined using the Folin–Ciocalteu colorimetric method [51], with some modifications. For that, 10 µL of propolis solution (50 to 300 µg/mL) was added to 40 µL of 7.5% (*w*/*v*) Na_2_CO_3_ and 50 µL of 10% (*w*/*v*) Folin–Ciocalteu reagent, all in absolute ethanol. The mixtures were incubated for 1 h in the dark at room temperature and the absorbance read at 760 nm against a blank (EtOH). Gallic acid standard solutions (1–50 µg/mL) were used to obtain the calibration curve, and the TPC of the A16.EE was expressed as mg of gallic acid equivalents per gram of dry extract (mg GAE/g).

#### 2.3.2. Total Flavonoids Content

Total flavonoids content (TFC) in A16.EE was estimated using an adaptation of the method of Woisky and Salatino [52], adding 50 µL of 2% (*w*/*v*) AlCl_3_ in EtOH to 50 µL extract solutions (400–1800 µg/mL). After 1 h of incubation, protected from light at room temperature, absorbance was measured at 420 nm. The TFC of A16.EE was calculated by comparison with the standard quercetin (5–200 µg/mL) and expressed as milligrams of quercetin equivalents per gram of dry extract (mg QE/g).

#### 2.3.3. Total *Ortho*-Diphenols Content

The total *ortho*-diphenols content (TOC) was evaluated through an adaptation of the method described by Durán et al. [53]. Briefly, 160 µL of A16.EE (150–300 µg/mL) was mixed with 40 µL of 5% (*w*/*v*) Na_2_MoO_4_ prepared with EtOH 50% (*v*/*v*). Mixtures were incubated for 15 min at room temperature in the dark and absorbance measured at 370 nm. As for TPC, gallic acid was used as the standard and TOC of A16.EE was expressed as mg GAE/g.

### 2.4. In vitro Activity of A16.EE against Phytopathogenic Fungi and Oomycetes

#### 2.4.1. Fungi and Oomycete Strains, Media and Growth Conditions

Strains of filamentous fungi and the oomycete used to assess propolis antifungal activity (Table 1) were grown in PDA medium (Thermo Fisher Scientific Inc., Waltham, MA, USA), at 24 °C (±1 °C), in the dark. Ground olive leaves were added to PDA (4 g/L) for *Colletotrichum fioriniae* growth.

#### 2.4.2. Evaluation of A16.EE Antifungal and Anti-Oomycetal Activities

In vitro antifungal and anti-oomycetal activities of the propolis were assessed according to an adaptation of the agar dilution method as described by Gallez et al. [54]. Prior to solidification, 50 µL of A16.EE solutions were mixed with 20 mL of PDA medium to final concentrations of 500, 1000 and 2000 µg/mL and immediately poured into Petri dishes. PDA medium alone and PDA with 50 µL of absolute ethanol were used as controls (C-H_2_O and C-EtOH, respectively). After solidification, a 6 mm diameter mycelial disc from the actively growing margin of a 5- to 10-day-old pure culture of filamentous fungus or oomycete was placed at the centre of the PDA plates. The cultures were incubated at 24 °C (±1 °C), in the dark, until the mycelia reached the edge of Petri dish or stopped growing. Two perpendicular lines intersecting the centre of the inoculum were drawn at the bottom of the plates and the 4 radii were measured once or twice a day, depending on mycelial growth speed (with measurements at least 9 h apart), to calculate the average mycelial diameter. The macroscopic characteristics of the mycelia were examined and registered daily.

#### 2.4.3. Gompertz Model Applied to Filamentous Growth

The Gompertz nonlinear regression model was used to fit and describe mycelial radial growth along time using Equation (2), adapted by Tjørve and Tjørve [47]:

(2) $$Y\left(t\right)=Y_{\max}\;\times \;\exp\left(-\exp\left(-{\;k}_{G}\left(t-t_{i}\right)\right)\right)$$

where *Y*(*t*) is the mycelium diameter (mm) as a function of time, *t* is time (days), *Y_max_* (mm) represents the upper asymptote (maximum diameter reached by the mycelium), *k_G_* is a growth-rate coefficient ((*Y*/*Y_max_*)/*t*, days^−1^), which affects the slope of the tangent line at the inflection point, and *t_i_* is the time at inflection (days), corresponding to the time that the mycelium reaches its maximum growth rate. The specific growth rate (μ) (mm/day) was calculated with Equation (3):

(3) $$µ=Y_{\max}\;\times \;k_{G}/e$$

where *e* is the Euler’s number. For each culture and condition, the mycelial diameter as a function of time was fitted to the Gompertz function, the respective growth curves were plotted and the growth parameters (*Y_max_*, *t_i_* and *μ*) were calculated. The inhibitory effect of each treatment was also calculated as the percent mycelial growth inhibition compared to the control at the day that the latter colonized the entire plate area, following Equation (4):

(4) $$\mathrm{Inhibition}\;\mathrm{of}\;\mathrm{mycelial}\;\mathrm{growth}\;\left(\%\right)\;=\;(D_{C}\;{-\;D}_{T})/D_{C}\;\times \;100$$

where *D_C_* is the average diameter of the control and *D_T_* is the diameter of each replicate colony under treatment at the same time point.

### 2.5. Statistical Analysis

The results are presented as the mean ± standard deviation (SD). For antifungal and anti-oomycetal assays, three independent experiments were carried out and, in the case of the chemical characterization of A16.EE, there were three independent experiments each with three replicates. Data were analysed using one-way analysis of variance (ANOVA) followed by a post hoc Tukey test for multiple comparisons. The statistical significance of differences between mean values is represented by the lettering notation; means followed by at least one same letter were not significantly different (*p* > 0.05). All statistical analyses and graphs were made with GraphPad Prism version 7.0.0 for Windows (GraphPad Software, San Diego, CA, USA), www.graphpad.com (accessed on 28 November 2023).

## 3. Results

### 3.1. Extraction Yield and Chemical Characterization of A16.EE

The A16 extraction yield and the mean content in total phenolics (TPC), total flavonoids (TFC) and total *ortho*-diphenols of the extract obtained—A16.EE—are depicted in Table 2.

### 3.2. Quantitative Characterization of the Antimicrobial Activity of Propolis Extract against Phytopathogenic Filamentous Species

**Figure 1:**
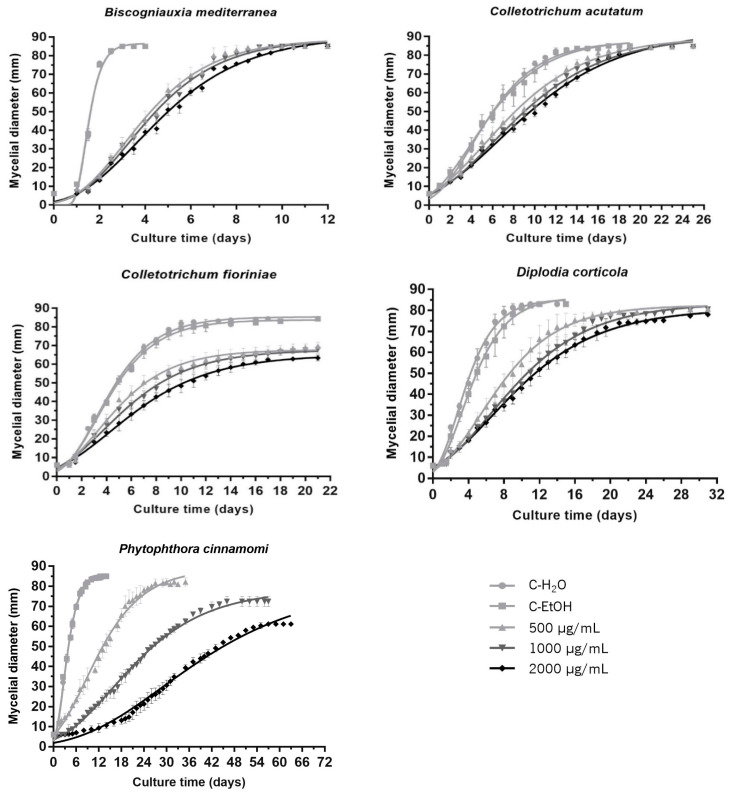
Growth curves estimated using the Gompertz mathematical model for *Biscogniauxia mediterranea*, *Colletotrichum acutatum*, *Colletotrichum fioriniae*, *Diplodia corticola* and *Phytophthora cinnamomi* cultures in PDA medium (●) and PDA supplemented with A16.EE reconstituted in absolute ethanol for final concentrations of 500 (▲), 1000 (▼) and 2000 μg/mL (◆). The control (■) was prepared with 50 µL of absolute ethanol (C-EtOH). Each curve represents the mean of three independent experiments, and error bars the respective SD.

In the first analysis, two main results stood out: the fit of the experimental data to the Gompertz equation was very good for all growth curves (r^2^ > 0.98), and the three concentrations of A16.EE tested were able to inhibit the mycelial growth of all the fungi and oomycete studied significantly in vitro (Figure 1; Table 3). Although A16.EE exerted an inhibitory effect on the colony growth, the response to increasing concentrations of the propolis extract was different (Figure 1). Just by observation, it is possible to distinguish two types of response regarding the concentration effect, dependent and independent, and two types regarding achieving (or not) the maximum potential growth, similar to the control or a premature growth stop (Figure 1). Quantitatively (Table 3), the estimated parameters translate these two dichotomies very well and further discriminate responses between species and the stringency of the concentration dependency. From Table 3, it is possible to verify that for *B. mediterranea*, *C. acutatum* and *D. corticola*, no significant differences were observed in the specific growth rates (μ) of the cultures when comparing the three A16.EE concentrations; instead, *C. fioriniae* and *P. cinnamomi* showed a consistent decrease in μ, with emphasis for the oomycete.

Unlike *μ*, the inflection time (*t_i_*) varied (increased) in a concentration-dependent manner for all the species, reflecting the delay in colony growth when in the presence of A16.EE (Table 3). For the highest concentration tested, the *t_i_* value for *B. mediterranea* and *P. cinnamomi* cultures was, respectively, 2.7× and 8.8× higher than in the absence of the extract (C-EtOH). *D. corticola*, *C. acutatum* and *C. fioriniae* had a less pronounced increase, with t_i_ values of 2.1×, 1.7× and 1.4× higher, respectively. The profile of the growth curves and the reported t_i_ increase seemed to demonstrate that A16.EE exerts its inhibitory effect from an early stage of mycelial growth. These results suggest that this parameter is more sensitive, having higher discriminant power than μ.

Regarding the maximum colony growth (*Y_max_*) reached in the presence of propolis extract, the two behaviours referred to above were also clearly distinguished by this parameter (Table 3). Thus, while *B. mediterranea* and *C. acutatum* ended up filling the entire area of the plate for the three concentrations tested (Figure 1), *C. fioriniae*, *D. corticola* and *P. cinnamomi* revealed a significant decrease in *Y_max_* for all concentrations tested, with the exception of *P. cinnamomi* at 500 µg/mL (Table 3). *P. cinnamomi* stood out by taking the longest time to reach maximum growth, and was the smallest one (smaller colonies), and was also the most responsive species regarding the range of concentrations tested. This disparity in relation to the fungi species’ responses was captured by the model being translated into the highest relative variations in all the growth parameters analysed (Table 3).

**Figure 2:**
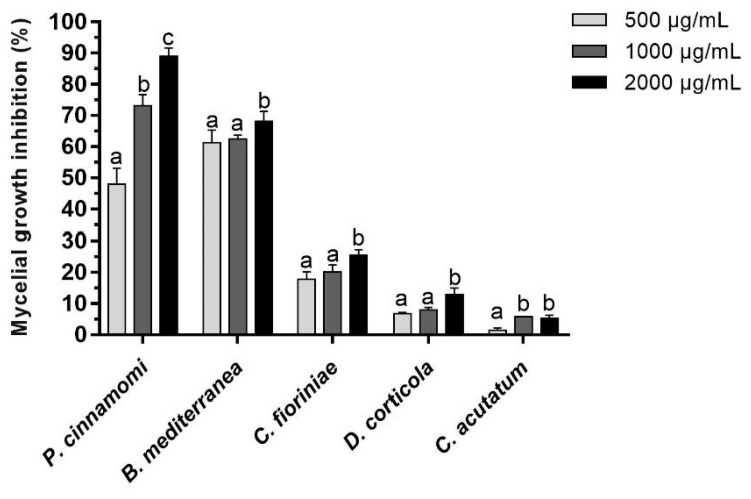
Mycelial growth inhibition of *Biscogniauxia mediterranea*, *Colletotrichum acutatum*, *Colletotrichum fioriniae*, *Diplodia corticola* and *Phytophthora cinnamomi* in the presence of 500 (■), 1000 (■) and 2000 (■) μg/mL of A16.EE, calculated in relation to growth in C-EtOH at the day that mycelia covered the plate’s entire area. Each bar represents the mean of three independent experiments, and error bars the respective SD. Statistical notation: for each species, mean values with the same letter were not significantly different (*p* > 0.05).

The percentage of mycelial growth inhibition was determined for all the species and concentrations tested, at the day mycelia covered the entire area of the control plate (Figure 2).

*B. mediterranea* and *P. cinnamomi* were clearly the most susceptible strains to A16.EE, and *C. acutatum* the least susceptible. At 500 μg/mL, *B. mediterranea* showed the highest growth inhibition (61.37%), followed by *P. cinnamomi* (48.04%). At 1000 and 2000 μg/mL, *P. cinnamomi* growth was inhibited by 73.14 and 89.02%, respectively, while *B. mediterranea* showed inhibitions similar to those observed with 500 μg/mL (62.55 and 68.24%, respectively), emphasizing the different behaviours of these two species regarding the dependence towards A16.EE concentration. In turn, *C. acutatum* showed inhibitions of mycelial growth below 6% for the three concentrations tested, while *D. corticola* and *C. fioriniae* registered inhibitions between 6.82 and 12.98% and between 17.84 and 25.49%, respectively. For *P. cinnamomi*, very significant differences were observed when comparing the three concentrations of A16.EE, in line with those already described for this species in the growth parameter analyses (Table 3).

**Figure 3:**
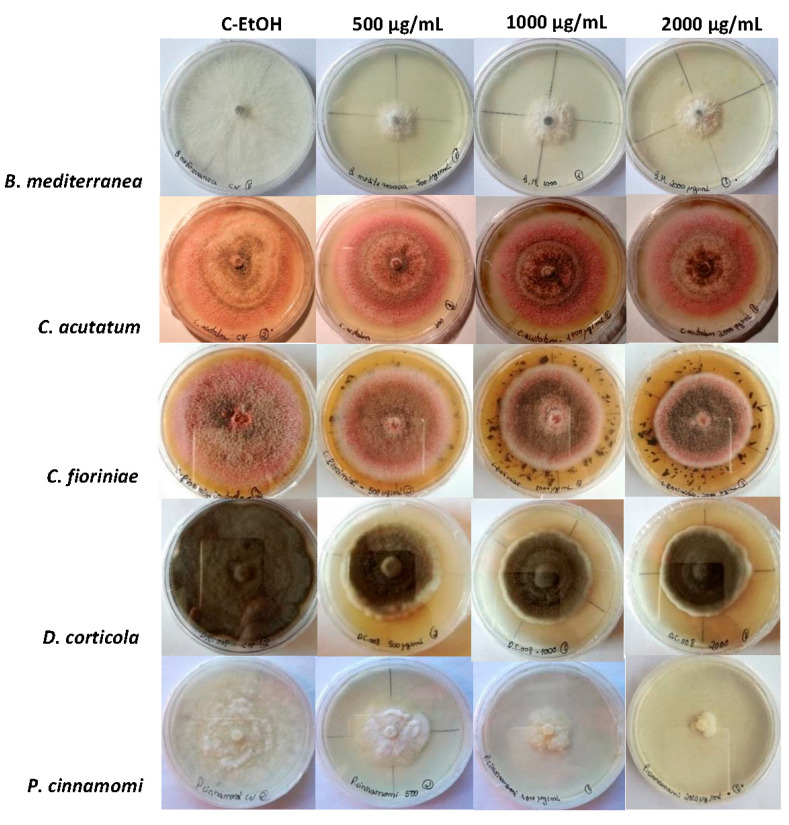
Macroscopic aspects of *Biscogniauxia mediterranea*, *Colletotrichum acutatum*, *Colletotrichum fioriniae*, *Diplodia corticola* and *Phytophthora cinnamomi* cultures at PDA medium supplemented with 500, 1000 and 2000 μg/mL A16.EE. Control (C-EtOH) was prepared with ethanol.

Regarding the qualitative macroscopic analysis, no noticeable changes were detected in the characteristics of *B. mediterranea*, *D. corticola* and *P. cinnamomi* cultures grown in the presence of propolis (Figure 3). However, the central part of *C. acutatum* and *C. fioriniae* colonies showed a more intense brown colour in the presence of A16.EE (at all concentrations) than in its absence.

## 4. Discussion

### 4.1. A16 Shows Chemical Characteristics from Propolis Typical from the South and North of Portugal, Suggesting a Gradient Is More Likely than a Dichotomy

The ethanolic extraction yield of sample A16 fits within the range of yields obtained by our research group for Portuguese propolis samples being closer to the upper limit (61.6% to 96.4%) (from a collection of unpublished studies of our investigation group). Lower yield values for propolis samples from the north of Portugal—61.6% [34] and 62% [55]—were found. Similarly, the yield reported in this study is higher in comparison to the values of ethanol extracts from propolis samples of other countries, such as from China (51.03%) [56] and from Taiwanese green propolis (66.75%) [57]. The results of TPC and TFC characterization are in line with the values reported by Silva et al. [58] for an EE from Beja (south of Portugal), but are also comparable to those of other European countries such as Croatia [59]. A16.EE showed a lower TPC in comparison to propolis samples from the north and centre of Portugal (characterized by a dark orange colour and being hard and brittle), which ranged between 202.3 and 415.5 mg GAE/g extract [25,36,58,60], and from other European regions such as the Basque Country (200–340 mg GAE/g extract) [61,62]. Regarding TFC, A16.EE presents similar content to that of an EE from the interior centre of Portugal (30.21 mg QE/g of extract), although showing a lower TPC than this extract (160.4 mg GAE/g extract) [25]. Also, the presence of *Cistus ladanifer* L. around the apiary may suggest A16 fitting in the less common Portuguese propolis type—typical of the central interior and south of the country, elastic and gummy, with dark green colour such as A16 and less rich in phenolics—for which a TPC range between 54.8 and 189.1 mg GAE/g extract was reported [36,63]. However, TPC and TFC values of A16.EE far exceeded the minimum contents proposed by Falcão et al. [36] as quality standards of the less common Portuguese propolis (6 and 2%, respectively). Together, these results corroborate the idea that intermediate profiles can be found, especially in areas where some overlap/gradient in botanical species may exist. Indeed, a previous study with an extract from Caramulo (interior centre of Portugal) also observed a similar result, noting that a “diversity and some deviation from the reference values can occur even in the same region/country, probably due to the availability of botanical species for propolis production in a given local and time” [64]. TPC and TFC in A16.EE are lower than those reported for Caramulo extract (166.3 mg GAE/g extract and 69.3 mg QE/g extract, respectively) [64]. Unlike for a wide variety of plant extracts [65,66], just one study was found determining TOC in propolis [34]. However, we consider this analysis essential since the *ortho*-diphenols is the class of phenolic compounds with the highest antioxidant power [67,68,69], proposing the inclusion of this assay in the common set of tests performed with propolis samples. Freitas et al. [34] reported a lower value for a propolis extract from the propolis from the north of Portugal (263.05 ± 15.19 mg GAE/g extract) than the value found in our study.

### 4.2. In Vitro Activity of Propolis against Phytopathogenic Filamentous Species

Several authors have corroborated the promising potential of propolis extracts against phytopathogenic filamentous fungi/oomycetes [70,71,72,73,74]. However, due to lack of uniform methodologies for the evaluation and characterization of antimicrobial effects, a fair comparison of efficiencies is generally impeded. Loebler et al. [73] reported that a propolis hydroalcoholic (ethanol 70%) extract from the centre of Portugal, at 100 μg/mL, inhibited 30% of the *Colletotrichum gloeosporioides* mycelial growth after ten days of inoculation, while 500 and 1000 μg/mL induced inhibitions of about 70 and 80%, values much higher than those described here for species of the *Colletotrichum* genus. Moreover, 100 to 1000 μg/mL of this extract inhibited between 50 and 80% of *Botrytis cinerea* growth, respectively [73]. Pereira et al. [64] obtained inhibitions of *Penicillium expansum*, after four days of inoculation, of 36, 42 and 48% for 500, 1000 and 2000 μg/mL for a propolis hydroalcoholic extract (ethanol 70%) from the centre of Portugal, respectively. Yusuf et al. [70] reported that 3 to 10 μg/mL of a methanol extract of Turkish propolis, with an incubation period of four days, completely inhibited the growth of *Phytophthora infestans*, *Phytophthora capsici* and *Phytophthora parasitica*. The latter result, in line with ours, gives support to the hypothesis of oomycetes, at least of the *Phytophthora* genus, having a much higher susceptibility towards propolis than true filamentous fungi. García et al. [75] observed an approximately 35% mycelial growth inhibition of *C. acutatum* for a Colombian propolis ethanol extract at 500 and 1000 μg/mL after four days of inoculation. To compare our results obtained for *C. acutatum* (inhibitions of less than 6%) with those of García et al. [75], the percent inhibition of *C. acutatum* by A16.EE was calculated for the fourth day of growth, and similar results were obtained with 500 and 1000 μg/mL A16.EE—25.96 (±8.81) and 28.60 (±3.95) %, respectively.

The mechanisms of action of propolis against filamentous fungi have not yet been fully elucidated. Some of the bioactive compounds from propolis extracts seem to induce a reduction in the ergosterol biosynthesis, leading to a loss of cell membrane integrity [74,76]. It is also known that they may inhibit the enzyme 1,3-β-glucan synthase, with consequent interference in the synthesis of 1,3-β-glucan, and they demonstrate an ability to bind to chitin, affecting fungal cell wall integrity [74,76]. Yang et al. [77] reported a high inhibitory activity of an EE of Chinese propolis against *Penicillium italicum*, having identified pinocembrin, pinobanksin, chrysin and galangin as the compounds probably responsible for such activity. Indeed, pinocembrin isolated from a Chinese propolis sample exhibited strong activity against *P. italicum*, with Peng et al. [78] concluding that this compound inhibits cellular respiration, causing an imbalance in mitochondrial homeostasis with a consequent disruption in cell membranes and metabolic disorders in the pathogen. Also, Chudapongse et al. [79], in a study with the human pathogenic yeast *Candida albicans*, pointed out that galangin inhibits NADH oxidation and CCCP (carbonyl cyanide m-chlorophenyl hydrazone)-stimulated respiration in isolated mitochondria, thus suggesting a galangin interference with the electron transport chain of this fungi. A study with propolis from Brazil observed that the antifungal activity against *C. albicans* was due to induced apoptosis and the disruption of the expression of genes involved in crucial processes such as cell adhesion, biofilm formation and filamentous growth [80]. Prenyl caffeate isolated from French propolis extracts showed significant antifungal activity against *Candida glabrata* and *C. albicans* (MIC_80_ of 16 and 62 µg/mL, respectively); pinocembrin had moderate activity (MIC_80_ 62–125 µg/mL), and pinobanksin-3-acetate, chrysin, and galangin exhibited weak activity (MIC_80_ ≥ 250 µg/mL) [81]. Agüero et al. [82] isolated and characterised bioactive compounds from methanolic extracts of propolis from Argentina, with 2’,4’-dihydroxy-30’-methoxychalcone and 2’,4’-dihydroxychalcone being the main antifungal compounds.

The colour alteration in *C. acutatum* and *C. fioriniae* colonies in the presence of the extract possibly reveals the occurrence of metabolic alterations [83]. Not excluding other causes, since information regarding this aspect is scarce, the brown colour of these colonies could be due to a class of dark/brown pigments called melanins, which are secondary metabolites produced by fungi via the oxidative polymerisation of phenolic compounds. It was reported that these pigments contribute to survival under environmental stress conditions [84,85]. Indeed, *Colletotrichum* species are known to produce DHN melanins [84,86,87,88].

In the case of sterol-auxotroph oomycete *Phytophthora cinnamomi*, compounds from propolis extracts may possess sterol-binding activity, possibly by acting as a competitor of sterol sensors or by sequestration sterols from the membrane, inhibiting the growth of this oomycete [89,90]. They may also attack 1,3-β-glucan, which are a major component of oomycete cell walls [91]. Miljanović et al. [92] observed a strong activity of an EE of propolis from Croatia against the pathogenic oomycetes *Aphanomyces astaci* and *Saprolegnia parasitica*, having predicted apigenin, chrysin and pinocembrin as the compounds responsible for this activity. With oomycetes being amongst the most important plant pathogens causing plant diseases worldwide [93], this particular susceptibility of oomycete species to propolis extracts and isolated compounds also constitutes a unique experimental tool, with potential high impact in plant disease control. In fact, *P. cinnamomi* susceptibility to A16.EE stood out, but as the only oomycete of the microbial panel, to know if this particular behaviour/susceptibility to propolis extract is characteristic of this class of filamentous microorganisms, further investigation will be needed. This is especially relevant since several plant extracts with antifungal activity exhibit less efficiency against *Phytophtora cinnamoni* than true filamentous fungi [94].

### 4.3. Gompertz-Derived Parameters Are Robust Descriptors and Exhibit High Discriminating Power

Permana et al. [48] and Camenzind et al. [49] applied a different parameterisation of the Gompertz model for modelling filamentous fungal growth. As far as we know, the parameterisation of the model proposed herein to describe the growth profiles of filamentous fungi/oomycetes cultures on solid media is a pioneering approach. Results clearly showed that it is a useful analysis tool, particularly in the context of toxicity assays, since it allowed a quantitative characterisation of mycelial radial growth and of the antifungal/anti-oomycetal effect of antimicrobial agents as the propolis extract. The three growth parameters analysed were concentration- and species-sensitive, revealing a high discriminating power, very useful both in screening and secondary assays.

## 5. Conclusions

We have demonstrated that the Gompertz equation accurately described the profile of mycelial growth of filamentous fungi/oomycetes colonies in solid media, and its estimated and derived parameters allow for a high discriminating power in antifungal/anti-oomycetal assays. Under the conditions of this study, the whole range of concentrations tested was able to reduce the in vitro growth of four economically important phytopathogenic fungi and an oomycete, with *B. mediterranea* and *P. cinnamomi* being the most susceptible to A16.EE. These findings support the potential role of A16.EE as a biopesticide in disease management programs, and thus its potential to reduce the use of synthetic fungicides, although future work would be required to validate the antifungal action of propolis in the field under natural environmental conditions. It is crucial to test the effective A16.EE concentrations in bioassays with the adequate explants (depending on the fungal strain and mode of infection) from susceptible plants species in order to identify the phytotoxicity threshold of A16.EE. After ex situ experiments under controlled conditions were carried out, field experiments could be more rationally designed to validate the effects in relevant crop species. Also, if particular extracts exhibited high phytotoxicity, good perspectives are still open for A16.EE as a source of bioherbicides. These studies are vital to promote propolis as a source of bioproducts, due to the introduction of numerous new legislatives provisions and guides to register newly formulated biopesticides.

The use of propolis In agronomic applications is still poorly explored. To our knowledge, this study is the first evidence for the activity of propolis against *B. mediterranea*, *C. fioriniae*, *D. corticola* and *P. cinnamomi*, contributing to its acknowledgement as a high-value natural product and to the socio-economic dynamisation of the Portuguese beekeeping sector.

## Figures and Tables

**Table 1:** Strains of filamentous fungi and the oomycete used to test antifungal/anti-oomycetal activities of the propolis extract. Strains were cultivated in PDA medium at 24 °C, in the dark, and periodically re-cultivated to new PDA medium-containing plates.

Filamentous Fungi/Oomycete	Reference	Environmental Isolates	Origin
*Biscogniauxia mediterranea*	Br41	*Quercus suber* L.	Teresa Lino Neto (University of Minho)
*Colletotrichum acutatum*	PT227	*Olea europaea* L	Rui Oliveira (University of Minho)
*Colletotrichum fioriniae*	unknown	*Olea europaea* L.	Paula Baptista (Polytechnic Institute of Bragança)
*Diplodia corticola*	CAA008	*Quercus suber* L.	Rui Oliveira (University of Minho)
*Phytophthora cinnamomi*	PH107	*Castanea sativa* Mill.	Rui Oliveira (University of Minho)

**Table 2:** Ethanolic extraction yield obtained from A16. Total phenolics content (TPC), total flavonoids content (TFC) and total *ortho*-diphenols content (TOC) of the A16.EE.

Extract	Yield (%)	TPC (mg GAE/g Extract)	TFC (mg QE/g Extract)	TOC (mg GAE/g Extract)
A16.EE	83.4	89.58 ± 3.49	30.47 ± 1.01	352.25 ± 3.43

**Table 3:** Growth parameters estimated by the Gompertz mathematical model (*t_i_* and *Y_max_*) and calculated from Equation (3) (*μ*) after fitting growth data of *Biscogniauxia mediterranea*, *Colletotrichum acutatum*, *Colletotrichum fioriniae*, *Diplodia corticola* and *Phytophthora cinnamomi* cultures in PDA medium supplemented with 500, 1000 and 2000 μg/mL of A16.EE. Values represent the mean ± S.D. of three independent experiments. Statistical notation: for each species and parameter, mean values with common letters are not significantly different (*p >* 0.05).

*B. mediterranea*	*µ* (mm/day)	*t_i_* (days)	*Y_max_* (mm)
C-H_2_O	80.74 (±1.53) ^a^	1.37 (±0.03) ^a^	86.47 (±0.15) ^a^
C-EtOH	79.90 (±1.67) ^a^	1.36 (±0.03) ^a^	86.51 (±0.08) ^a^
500 µg/mL	15.01 (±0.92) ^b^	3.19 (±0.16) ^b^	85.42 (±1.91) ^b^
1000 µg/mL	14.51 (±0.35) ^b^	3.31 (±0.03) ^bc^	85.34 (±1.96) ^b^
2000 µg/mL	12.90 (±0.46) ^b^	3.61 (±0.20) ^c^	85.21 (±0.94) ^b^
*C. acutatum*	*µ* (mm/day)	*t_i_* (days)	*Y_max_* (mm)
C-H_2_O	9.84 (±0.19) ^a^	4.07 (±0.07) ^a^	86.10 (±0.17) ^a^
C-EtOH	9.05 (±1.45) ^a^	3.86 (±0.45) ^a^	86.48 (±1.23) ^a^
500 µg/mL	6.38 (±0.10) ^b^	5.35 (±0.55) ^b^	85.70 (±1.04) ^b^
1000 µg/mL	6.01 (±0.13) ^b^	6.02 (±0.28) ^bc^	85.51 (±1.05) ^b^
2000 µg/mL	5.50 (±0.24) ^b^	6.44 (±0.32) ^c^	85.33 (±0.66) ^b^
*C. fioriniae*	*µ* (mm/day)	*t_i_* (days)	*Y_max_* (mm)
C-H_2_O	11.78 (±0.40) ^a^	3.11 (±0.09) ^a^	85.13 (±0.10) ^a^
C-EtOH	11.22 (±1.10) ^a^	3.14 (±0.09) ^a^	84.10 (±0.66) ^a^
500 µg/mL	7.54 (±0.89) ^b^	3.43 (±0.24) ^a^	68.77 (±2.28) ^b^
1000 µg/mL	6.30 (±0.65) ^bc^	3.75 (±0.33) ^ab^	68.14 (±1.93) ^b^
2000 µg/mL	5.24 (±0.61) ^c^	4.35 (±0.45) ^b^	65.09 (±0.71) ^c^
*D. corticola*	*µ* (mm/day)	*t_i_* (days)	*Y_max_* (mm)
C-H_2_O	13.69 (±1.23) ^a^	2.78 (±0.12) ^a^	85.33 (±0.17) ^a^
C-EtOH	11.77 (±1.46) ^a^	3.23 (±0.30) ^a^	86.32 (±0.30) ^a^
500 µg/mL	6.45 (±0.94) ^b^	5.53 (±0.67) ^b^	81.10 (±1.67) ^b^
1000 µg/mL	5.06 (±0.51) ^b^	6.66 (±0.38) ^bc^	80.94 (±1.26) ^b^
2000 µg/mL	4.54 (±0.42) ^b^	6.83 (±0.57) ^c^	78.81 (±0.83) ^c^
*P. cinnamomi*	*µ* (mm/day)	*t_i_* (days)	*Y_max_* (mm)
C-H_2_O	14.47 (±0.32) ^a^	2.62 (±0.12) ^a^	86.68 (±0.31) ^a^
C-EtOH	14.15 (±0.64) ^a^	2.58 (±0.06) ^a^	86.03 (±0.57) ^a^
500 µg/mL	3.90 (±0.24) ^b^	8.54 (±1.14) ^ab^	90.23 (±1.41) ^a^
1000 µg/mL	2.22 (±0.13) ^c^	14.86 (±1.11) ^b^	78.91 (±2.72) ^b^
2000 µg/mL	1.55 (±0.12) ^c^	22.67 (±5.12) ^c^	68.68 (±2.34) ^c^

## Data Availability

Data are contained within the article.
